# Analysis of Traffic Signs Information Volume Affecting Driver’s Visual Characteristics and Driving Safety

**DOI:** 10.3390/ijerph191610349

**Published:** 2022-08-19

**Authors:** Lei Han, Zhigang Du, Shoushuo Wang, Ying Chen

**Affiliations:** School of Transportation and Logistics Engineering, Wuhan University of Technology, Wuhan 430063, China

**Keywords:** traffic signs, TSIV, simulation, visual characteristics, visual workload intensity, driving safety

## Abstract

To study the influence of traffic signs information volume (TSIV) on drivers’ visual characteristics and driving safety, the simulation scenarios of different levels of TSIV were established, and 30 participants were recruited for simulated driving tests. The eye tracker was used to collect eye movement data under three-speed conditions (60 km/h, 80 km/h, and 100 km/h) and different levels of TSIV (0 bits/km, 10 bits/km, 20 bits/km, 30 bits/km, 40 bits/km, and 50 bits/km). Principal component analysis (PCA) was used to select indicators sensitive to the influence of TSIV on the drivers’ visual behavior and to analyze the influence of TSIV on the drivers’ visual characteristics and visual workload intensity under different speed conditions. The results show that the fixation duration, saccade duration, and saccade amplitude are the three eye movement indicators that are most responsive to changes in the TSIV. The driver’s visual characteristics perform best at the S3 TSIV level (30 bits/km), with the lowest visual workload intensity, indicating that drivers have the lowest psychological stress and lower driving workload when driving under this TSIV condition. Therefore, a density of 30 bits/km is suggested for the TSIV, in order to ensure the security and comfort of the drivers. The theoretical underpinnings for placing and optimizing traffic signs will be provided by this work.

## 1. Introduction

As the most prevalent traffic engineering facility, traffic signs provide visual information and route assistance to drivers by using information such as words, symbols, and graphics. For efficient and safe traffic flow, traffic signs are essential. However, the arbitrary placement of traffic signs has become more prevalent as a result of the accelerated development of road building and the growing congestion of the road network. The unreasonable traffic signs information volume (TSIV), which includes both insufficient information and information overload, is the most noticeable issue among them. The driver may experience slower cognitive reaction times, decreased cognitive efficiency, and greater visual workload due to information overload [1,2]. Insufficient information can lead to greater reliance on the driver’s judgment and even poor coping mechanisms ranging from distraction to aggression. Both insufficient and overloaded information have a negative impact on driving safety [3]. Therefore, a reasonable and appropriate TSIV is conducive to drivers to complete their driving tasks and ensure safe driving.

Many factors, including the sign’s size, words, color, reflectivity, and angle of inclination, must be considered when designing and installing traffic signs, but the most crucial one is whether the driver will accept and use the information [1]. The ability of humans to receive information in a short time is limited [4]. When the TSIV is excessive, the driver’s short-term memory will rapidly get overloaded, making it difficult to complete driving tasks and threatening traffic safety. Additionally, drivers primarily rely on their vision to collect information when driving [5]. As a result, the drivers’ visual characteristics will be impacted by the overload or insufficiency of TSIV [6].

On the TSIV, academics have conducted a great deal of studies and produced a great deal of findings. Lyu et al. [7] found that the drivers’ reaction time increased with the increase of the TSIV and proposed that the information contained in individual sign should be kept at an appropriate level so that drivers have enough time to identify the signs and thus ensure driving safety. To ensure driving safety, Guo et al. [8] proposed that the information volume on expressway traffic signs should be limited to 194 bits and no more than seven pieces of information based on the analysis of various indicators. Xu et al. The authors of [9] made a comprehensive evaluation of the graphical variable message sign from five aspects: legibility speed, legibility distance, legibility time, comprehension accuracy, and subjective scoring, and suggested that the upper limit of the information displayed on the graphical variable message sign was five road names. Liu et al. [10] found that simpler contents and larger contrast between the background colors and foreground colors of traffic signs would make the human brain respond faster. Lyu et al. [11] found that driving workload was closely related to TSIV and that speed maintenance and lane deviation was significantly different at different cognitive workload levels. Liu [1] found that the TSIV had a significant impact on the visual search performance of drivers, and the more TSIV, the less the efficiency of the visual search was. Liu et al. [12] investigated the effect of the TSIV on driver’s recognition time and showed that the TSIV explains 61% of driver’s recognition time. The more information is conveyed by road traffic signs, the longer the recognition time. The threshold value of TSIV is 642 bits, and exceeding this value will result in information overload.

In studies of traffic signs and drivers’ visual recognition, cognition, and driving behavior, it is well established that traffic sign information affects the drivers’ visual characteristics, cognitive abilities, and driving performance. Liu et al. [13] analyzed the information transmission system of the traffic signs combination from the perspective of cognitive psychology, constructed its information transmission model, and explored the relationship between the driver identification time in the process of different traffic sign combinations. Schnell et al. [14] found that larger and brighter signs are more effective in delivering information to drivers, either by reducing information acquisition time or by improving transmission accuracy. In return, reducing sign viewing duration and improving reading accuracy may improve road safety. According to Borteorte et al. [15], road users do perceive and process information differently. These differences are influenced by factors like gender, age, and experience, and the TSIV has an impact on how safe they perceive the road to be. The drivers significantly reduced their speed to read four-line monolingual and four-line bilingual signs, which was accompanied by an increase in headway to the vehicle in front, according to research by Jamson et al. [16] on the impact of bilingual traffic guide signs on driver attention at various length and complexity levels. Through the simulated driving test, Huang et al. [17,18,19,20] examined how the complexity of diagrammatic guide signs affected the drivers’ eye movements and driving behavior, emphasized the need for evaluating and optimizing complex diagrammatic guide signs, and suggested the ideal design scheme for advanced guide signs on the urban expressway. The impact of the layout form and information volume of the intersection guidance signs on the driver’s driving behavior was examined by Wei et al. [21] and Yao et al. [22]. The guide signs’ quantitative evaluation results reveal that visual safety reduces as information volume on the signs rises. Topolšek et al. [23] compared and analyzed the visual performance differences of billboards and traffic signs between drivers of various ages and discovered that the number of roadside objects detected was not correlated with the driver’s age and that those drivers who noticed more traffic signs also paid more attention to visual advertising. Younger drivers considerably outperformed older drivers in terms of accuracy and response time, according to Ben-Bassat et al. [24]. The older drivers had an average reaction time that was roughly twice as fast as the younger drivers. However, neither group’s comprehension of traffic signs was impacted by the presentation style (with or without context).

The aforementioned literature concentrated more on the evaluation of a specific road section or one traffic sign and mainly examined the effects of variables such as the information volume, layout form, and setting form of traffic signs on the driver’s visual characteristics, cognitive ability, and driving behavior. The reasonableness of the density of TSIV in road networks, which is one of the beginning grounds for this research endeavor, has not, however, received much attention. The objective of this study is to identify the ideal TSIV range for safe driving by analyzing how the density of road TSIV affects the drivers’ visual characteristics and visual workload.

It is anticipated to aid in assessing the TSIV’s rationalism and offer a theoretical framework for the logical placement of traffic signs. Additionally, several questions were addressed:

(1) Which visual indicators of drivers are most significantly and sensitively affected by the TSIV?
(2) How does the different TSIV affect the visual characteristics and visual workload of drivers?
(3) What is the appropriate range of TSIV that will ensure the safety and comfort of driving?

## 2. Quantification of TSIV

The transmission and feedback of traffic information is achieved in large part by the use of traffic signs. The primary purposes of traffic signs are to notify drivers of the current traffic situation in a clear, timely, and appropriate manner; to direct and organize the flow of traffic; and to promote the safe, orderly, and efficient operation of vehicles. As a result, the safety of road operations directly depends on the logic of traffic sign information. Among these, the information volume of the traffic signs can have a major impact on the drivers’ cognitive function and workload while driving, meaning that different traffic signs will have varied information workloads for the drivers. As a result, we must first quantify the information contained in traffic signs.

Shannon first proposed the concept of information theory in 1948 and defined “information entropy” [8]. Information theory explains that the volume of information contained in a certain given background and condition is closely related to the probability of each element in the information [25]. The TSIV calculation formula is as follows, in accordance with Shannon Information Theory:

(1) $$H\left(X\right)=-\sum_{i=1}^{m}P\left(X_{i}\right)\log_{2}P\left(X_{i}\right),$$

where *H*(*X*) indicates the information volume of the traffic signs (bits), *m* is the total number of possible states for the event *X*, *X_i_* represents the *i* state of the event, *P*(*X_i_*) represents the probability of the *i* state.

Assuming that the event in each state occurs with the same probability, that is, *P*(*X_i_*) = 1/*m*, the calculation method of information volume is reduced to:

(2) $$H_{i}\left(X\right)=\log_{2}m,$$

According to the Chinese national standard “Road Traffic Signs and Markings” (GB5768-2022) [26], the information elements of traffic signs mainly include seven types: Chinese characters (3500 commonly used Chinese characters), English characters (26 English letters from A to Z), Arabic numerals (10 Arabic numerals from 0 to 9), geometric figures (six geometric shapes including circles, triangles, etc.), colors (11 colors including red, white, blue, etc.), pointing symbols (including 30 different direction pointing arrows or symbols), and graphic symbols (including 50 different types of graphic symbols). The information volume of different information elements can be calculated by the Formula (2).

The weight of the traffic sign information elements must be established because when drivers notice traffic signs while driving, their perception and attention to various types of traffic sign information and elements varies. The weight of seven different types of elements was ultimately determined using the Analytic Hierarchy Process (AHP), which was used to assess the significance of the traffic sign information elements [27]. This process involved 30 experienced drivers and 30 traffic engineers comparing the traffic sign information elements pair by pair. The consistency index (CI = 0.0238) and consistency ratio (CR = 0.0267 < 0.1) show that the results of the hierarchical analysis have satisfactory consistency and the weight values of each element are valid. Therefore, the information volume and weight of each traffic sign information element were obtained, and the results are shown in Table 1.

After comprehensively considering the information volume and weight of each element, the information volume of traffic signs can be calculated by the Formula (3):

(3) $$H\left(X\right)=\sum_{i=1}^{m}\left(\varepsilon_{i}H_{i}n_{i}\right)$$

where: *ε_i_* is the weight of *i* element, *H_i_* is the basic information volume of the *i* element, *n_i_* is the number of *i* element in traffic signs.

The information volume of traffic signs is quantified in this study using the aforementioned computation approach to produce different levels of TSIV, which are then utilized to examine the visual characteristics and driving workload of drivers at various TSIV levels.

## 3. Experimental Design

### 3.1. Participants

For this study, 30 participants—21 males and 9 females—were recruited based on the gender ratio of Chinese drivers (7:3 for men to women) [28]. Additionally, the number of participants was chosen using examples from previous similar studies [1,9,12] that followed customary procedures. Each participant has at least three years of driving experience and a Chinese statutory class “C” driver’s license. With a standard deviation of 7.6 years, the participants’ average age was 32, and their average amount of driving experience was 9 years, with a 5.4-year standard deviation. No one was color blind, and everyone had normal vision. Before the test started, each participant signed an “Informed Consent to the Test,” and they each received RMB 200 thereafter.

### 3.2. Apparatus

**Figure 1:**
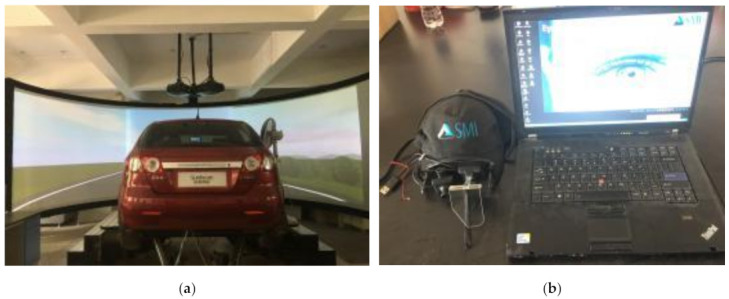
Apparatus of study. (**a**) The driving simulator; (**b**) the eye tracker.

The motion-based driving simulator used in this study has six degrees of freedom (DOF), as illustrated in Figure 1 (produced by OKTAL-SE, France). As a test vehicle, a real BYD F3 model automobile was employed. The driving simulator has a vision system with three projectors that projects the road model onto a spherical drape in front of the car, giving the driver a 300-degree horizontal field of view. The drivers can access actual roads, traffic signs and markings, and a variety of road environments through the driver simulator. The motion system, feedback system, and cockpit performance of the simulator have all improved to an advanced degree on a global scale.

The SMI iView XTM HED helmet eye movement tracking device, developed and manufactured by SMI (Senso Motoric Instruments) of Germany, was the eye tracker utilized in this examination. The driver’s whole of eye movement data while looking for and viewing various traffic environment details can be collected by the eye tracker. The eye tracker weighs 450 g, has a sampling frequency of 50 Hz or 200 Hz, a fixation position accuracy of 0.5° to 1.0°, and a tracking range of −30° to +30° and −25° to +25° in both the horizontal and vertical directions. In this study, the participants’ fixation, saccade, and blinking during the test were tracked using an eye tracker, and the data were recorded and saved on a laptop computer.

### 3.3. Driving Scenarios

First of all, the traffic signs designed in the test scenarios must comply with the current relevant standards in China, and the setting of the traffic signs must also meet the actual conditions and requirements of the road. More importantly, in order to fulfill the purpose of the experiment, the traffic signs set up in different sections should reflect the differences in the information volume.

The traffic signs set up in the test scenarios met the requirements of the relevant standards according to the actual conditions of the road. The traffic signs in the test scenarios were considered in terms of their type and structural form, in addition to the differences in information volume.

These traffic signs contained regulatory signs, warning signs, and guide signs. At the same time, the structural forms of the traffic signs included single vertical, double vertical, and cantilever structures. The diversity of forms of traffic signs was fully guaranteed. 

In order to determine the features of the kind, density, and distribution of the information volume on traffic signs along these highways, we performed field surveys on a number of China’s monotonous and non-monotonous highways. The distribution of TSIV varied significantly, as we discovered, and the findings were consistent with those of Lyu et al. [7]. The findings of the final investigation revealed that the TSIV increment was roughly 10 bits/km. Based on this, we assigned S0, S1, S2, S3, S4 and S5 as the five levels of the density distribution of TSIV in the test situations, and Table 2 displays the classification findings.

The following examples serve as illustrations of the simulated driving scenario created for this study:

(1) The secondary road in the driving simulation has one lane in each direction, a lane width of 3.75 m, and a designed speed of 80 km/h. The road’s design specifications all adhere to current Chinese regulations.
(2) The roads in the study’s simulated driving scenarios are all straight roads to prevent the impact of alignment changes on the experimental results.
(3) To prevent affecting the trial results, no traffic flow and a distinct scenery are set in the simulated driving environment.

**Figure 2:**
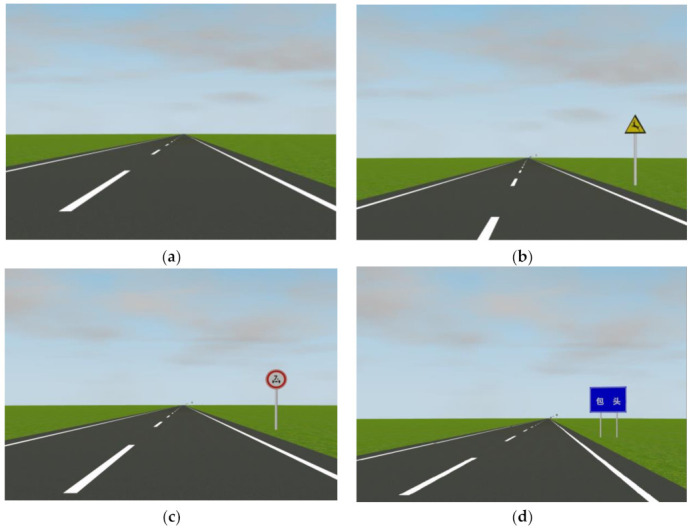
Part of traffic signs of simulated driving scenarios. (**a**) No traffic signs; (**b**) add warning signs; (**c**) add regulatory signs; (**d**) add guide signs.

The setting of part of traffic signs in the test scenarios is shown in Figure 2. It should be noted that the pictures displayed in Figure 2 only select representative types of traffic signs and do not represent the corresponding TSIV levels in the experiment because it is challenging to show all traffic signs of each level in one picture.

### 3.4. Experimental Procedure

The experimental design in the paper involved participants driving a simulated vehicle in a driving simulator, where participants were required to operate and maneuver the car at three speeds to complete the driving task. Then, we evaluated the visual characteristics of the drivers when recognizing different levels of TSIV under different speed conditions.

The road length in the simulated driving scenarios is about 20 km, and the duration for drivers to complete the tests at three different speeds was different, but the drivers will not experience driving fatigue during the tests to avoid affecting the test results.

The experimental process is as follows:

(1) An “Informed Consent Form” and a “Personal Information Registration Form,” which recorded the participant’s basic information, were distributed to each participant to read, sign, and submit.
(2) Started the system of simulating driving, ensured that each component was functioning properly, and fixed the simulated scenarios.
(3) Participants initially performed a 5- to 10-min adaptive driving simulation fitness test to determine whether participants were maladaptive. In such case, the participant was changed. If not, the formal test continued.
(4) The tester gave the participants an explanation of the simulation driving test’s procedure and safety considerations.
(5) Following proper preparation, the participants wore the eye tracker, made necessary adjustments and corrections, verified that the eye tracker was connected to the computer, and calibrated the eye tracker.
(6) The participant started the car, started the test, and drove at the specified speed while collecting data on eye movements.
(7) The participants went through three simulated driving tests in order, moving at speeds of 60 km/h, 80 km/h, and 100 km/h. The subject needed to maintain outstanding mental and physical health for 30 min following the conclusion of each experiment by resting or moving around.
(8) Following the completion of the test, the participant’s eye tracker was removed from the driving simulator and the data on their eye movements was preserved.
(9) Participants completed a subjective survey questionnaire.
(10) Replaced the participants and repeated the above steps.
(11) All the tests were completed.

**Figure 3:**
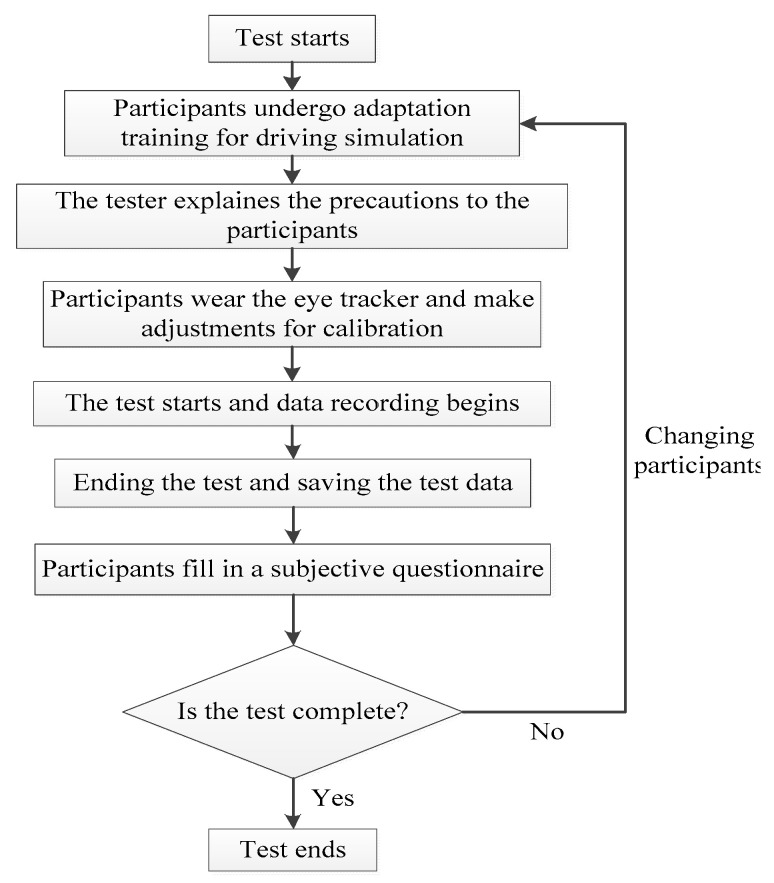
The process of simulated driving tests.

Figure 3 depicts how the paper’s simulated driving tests were conducted.

## 4. Selection of Eye Movement Indicators Based on PCA

The error of the eye movement data obtained in this trial is highly random [12], and therefore, the outliers in the eye movement data need to be removed to ensure the accuracy and rationality of the data analysis. In this paper, all anomalous data are removed using the method of PauTa Criterion [29,30], of which the basic formula is:

(4) $$\left|x_{i}-\bar{x}\right|>3\sigma ,$$

where: *x_i_* is each sample data; $\bar{x}$ indicates the average value of all the sample data; *σ* denotes the standard deviation of all sample data. Sample data outside this range were removed.

There will ineluctably be certain correlations among the numerous eye movement indicators of drivers, which will in turn cause the information indicated by these indicators to accumulate and overlap. Due to this, the established index evaluation system must eliminate the redundant and repeated information indicators in order to achieve the goal of reducing the dimension of the data, which is to reduce the number of linearly related indicators to a manageable number of irrelevant index systems.

The basic idea of principal component analysis (PCA) is to try to replace a large number of correlated indicators with a new set of uncorrelated composite indicators [31,32,33].

The following is a brief description of the calculation steps for PCA:

Suppose that there are n samples, each with *p* variables, and they form a matrix of order n × p.

(5) $$X=\left[\begin{array}{cccc} x_{11} & x_{12} & \cdots & x_{1p}\\ x_{21} & x_{22} & \cdots & x_{2p}\\ \vdots & \vdots & & \vdots \\ x_{n1} & x_{n2} & \cdots & x_{np} \end{array}\right],$$

(1) Calculation of the correlation coefficient matrix:

(6) R=r11r12⋯r1pr21r22⋯r2p⋮⋮⋮rp1rp2⋯rpp,

The correlation coefficient is calculated as follows:

(7) $$r_{ij}=\frac{\sum_{k=1}^{n}\left(x_{ki}-{\bar{x}}_{i}\right)\left(x_{kj}-{\bar{x}}_{j}\right)}{\sqrt{\sum_{k=1}^{n}{\left(x_{ki}-{\bar{x}}_{i}\right)}^{2}\sum_{k=1}^{n}{\left(x_{kj}-{\bar{x}}_{j}\right)}^{2}}},$$

(2) Solving for the eigenvalues and the eigenvectors.

Solving the eigenequations $\left|\lambda i-R\right|=0$, the eigenvalues are obtained, and it arranges the feature values in descending order $\lambda_{1}\geq \lambda_{2}\geq \cdots \geq \lambda_{p}\geq 0$; then, the eigenvector corresponding to the eigenvalues is solved: *e_i_*(*i =* 1, 2,…, *p*), and makes $\sum_{j=1}^{p}e_{ij}{}^{2}=1$.

(3) Calculation of principal component contribution rates and cumulative contribution rates.

The contribution rate of the sample principal component *Fi* is: $\lambda_{i}/\sum_{k=1}^{p}\lambda_{k}\left(i=1,2,\cdots ,p\right)$, the cumulative contribution of the principal component sample *F1*,…, *Fk* is $\sum_{k=1}^{i}\lambda_{k}/\sum_{k=1}^{p}\lambda_{k}(i=1,2,\cdots ,p)$.

Finally, the eigenvectors corresponding to eigenvalues with a cumulative contribution rate of 85% or more and an eigenroot greater than 1 are generally selected to form the principal components.

**Figure 4:**
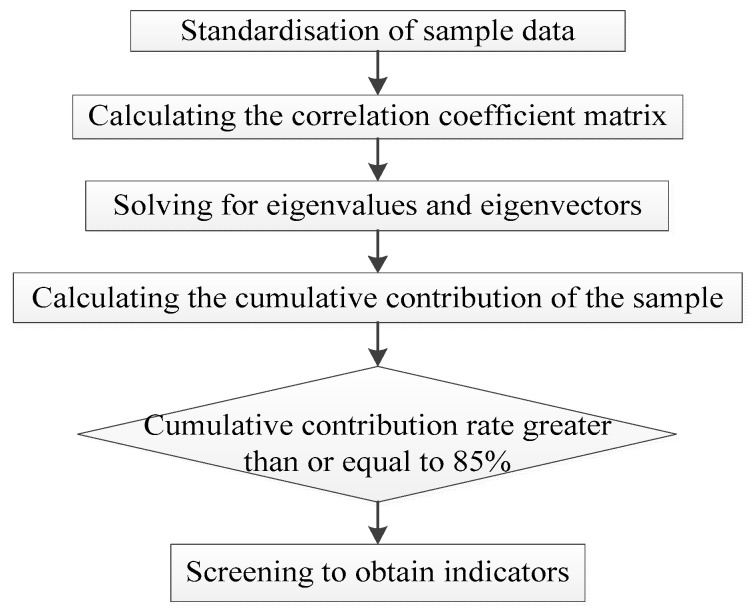
The process of selecting indicators by PCA.

The process of selecting indicators using PCA is shown in Figure 4.

The default Kaiser–Meyer–Olkin (KMO) test and the Barlett Test of Sphericity are chosen here to test the correlation between the original indicators before the PCA. The value of the KMO statistic ranged from 0 to 1, and the larger the value, the better the result of PCA. It is generally considered that the value of the KMO statistic less than 0.5 is not suitable for PCA. The Barlett Test of Sphericity is used to test whether the correlation coefficient matrix of the variables is a unit matrix. The test statistic obeys χ2, and if the test rejects the original hypothesis, i.e., *p* < 0.05, it is suitable for PCA.

Three frequently used indicators of driver’s visual behavior were chosen: fixation, saccade, and blink: blink duration (BD), fixation duration (FD), pupil diameter (PD), saccade duration (SD), saccade amplitude (SA), saccade peak velocity (SPV), and saccade average velocity (SAV). These indicators were chosen based on the requirements for PCA as well as the methods and findings of research on drivers’ visual behavior in the field of traffic safety. Additionally, standardizing these data, the correlation coefficient and *p*-value of the matrix was solved for the standardized data, and the KMO test and Barlett Test of Sphericity were done, the results of which are shown in Table 3 and Table 4.

Table 4 shows that the KMO statistic for the eye movement indicators is 0.548 > 0.5, and the result of the Barlett Test of Sphericity is *p* = 0.000 < 0.05, so the driver’s eye movement indicators are suitable for the PCA. The results of the PCA are shown in Table 5.

The eigenroots, the contribution rates, and the cumulative contribution rates of each principal component are ranked from largest to smallest in Table 5. The first principal component has an eigenvalue of 3.558 and a contribution rate of 50.825%, which explains 50.825% of the total variation, while the second principal component has an eigenroot of 1.459, explaining 20.846% of the total variation. The eigenroots of the first two principal components are greater than 1, and the cumulative variance contribution rate has reached 70.214%. Since the eigenroot of the third principal component is 0.991, which is close to 1, and the variance contribution rate is 14.154%, which is close to the second principal component, the cumulative contribution rate of the variance reached is 85.825%, that is, the three principal components are able to explain 85.825% of the total variance, indicating that the first three principal components essentially contained all the information available for the eye movement indicators. The principal component matrix for the eye movement indicators is shown in Table 6.

Thus, these three principal components can be expressed as:

F1 = −0.469BD − 0.497FD − 0.725PS − 0.082SD + 0.959SA + 0.727SPV + 0.623SAV,(8)

F2 = 0.618BD + 0.547FD − 0.114PS − 0.857SD − 0.134SA + 0.251SPV + 0.334SAV,(9)

F3 = 0.358BD + 0.807FD + 0.632PS − 0.321SD + 0.293SA + 0.075SPV − 0.110SAV,(10)

We define the first principal component as an evaluation indicator characterizing the driver’s search efficiency, the second principal component as an evaluation indicator of the driver’s capacity for information reception, and the third principal component as an evaluation indicator of the driver’s effort to acquire and perceive information by combining the meaning and weighting of each eye-movement indicator. The eye movement indicator with the highest weight in each principal component is chosen to replace the reduced dimensional composite indicator after taking into account the correlation between each eye movement indicator and each principal component. Finally, the three-eye movement sensitive indicators that are influenced by the TSIV throughout the driving process are identified as fixation duration (FD), saccade duration (SD), and saccade amplitude (SA).

## 5. Results and Analysis

### 5.1. Analysis of the Fixation Duration

**Figure 5:**
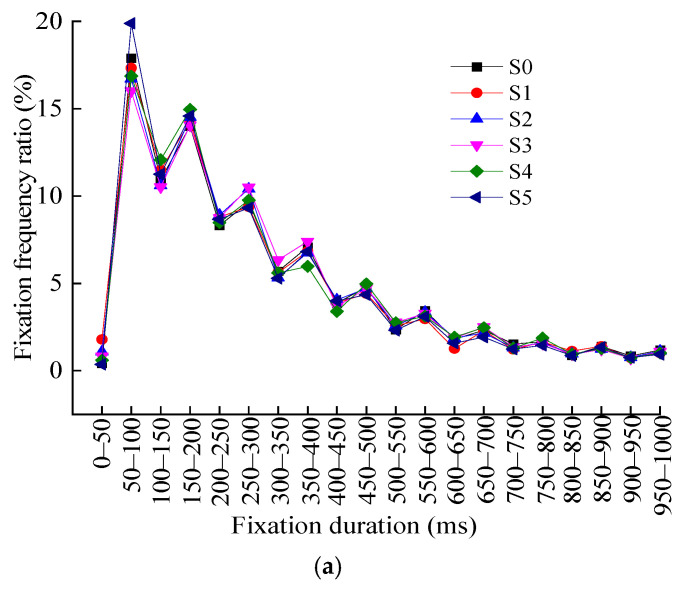

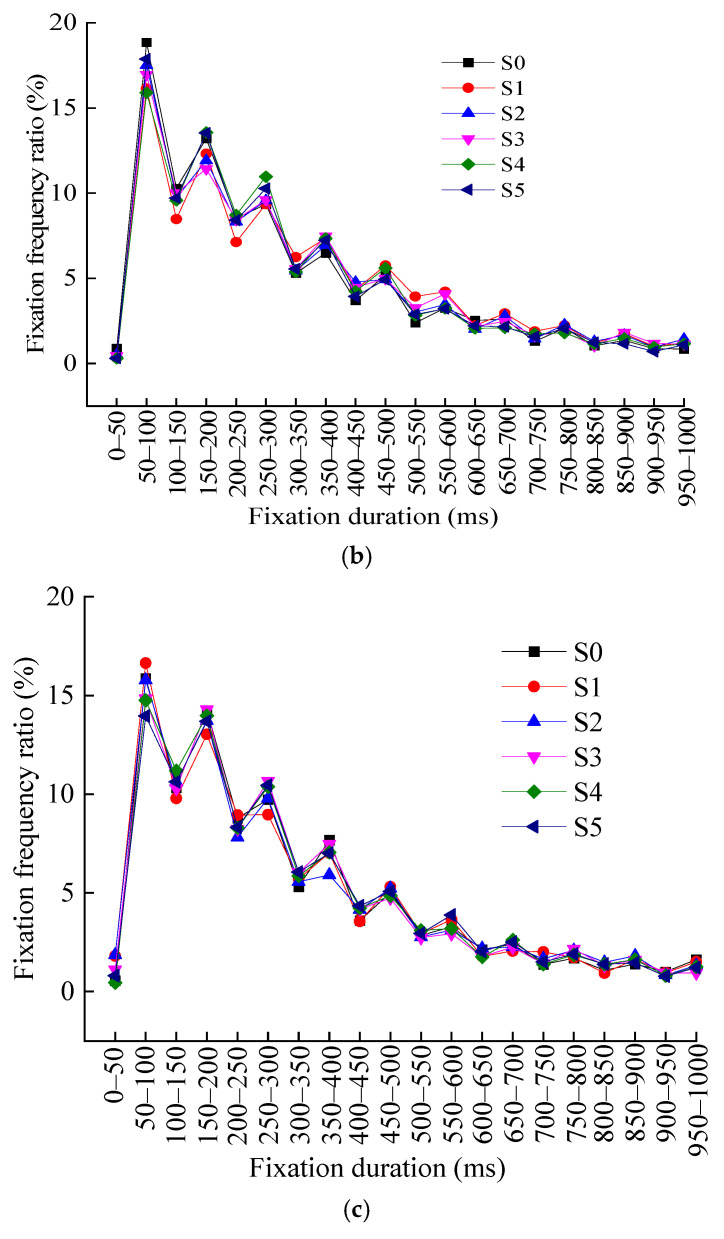
Distribution of driver’s fixation durations at different TSIV of the three speed conditions. (**a**) 60 km/h; (**b**) 80 km/h; (**c**) 100 km/h.

The fixation duration of the participants at three speeds of 60 km/h, 80 km/h, and 100 km/h was counted by an interval time period of 50 ms. Figure 5 shows the statistical results of the fixation duration and fixation frequency ratio when the driver recognizes the different levels of TSIV under the three-speed conditions, respectively.

From the statistical results of the driver’s fixation duration at the three-speed conditions of 60 km/h, 80 km/h, and 100 km/h, the following results can be obtained:

(1) Under the three-speed conditions, when recognizing different levels of TSIV, the frequency change law of fixation behavior in each fixation duration is highly consistent, showing a gradual downward trend of the driver in the fluctuation, and eventually tends to stabilize.
(2) The duration of the driver’s single fixation at different levels of TSIV under three-speed conditions is mostly concentrated in the range of 50–400 ms, with a fixation frequency of over 70%.
(3) The highest percentage of single fixation duration occurs in the time periods of 50–100 ms, 150–200 ms, and 100–150 ms, respectively, when drivers recognize different levels of TSIV under three-speed conditions, and the total percentage of fixation frequency in these three time periods exceeds 40%, indicating that drivers primarily perceive and obtain relevant information through the occurrence of shorter fixation behavior when recognizing different TSIV under three-speed conditions.
(4) When drivers recognize different levels of TSIV under three-speed conditions, the frequency of fixation behaviors with a single fixation duration of less than 50 ms and more than 800 ms is relatively low, indicating that the drivers’ visual recognition of information while driving rarely results in a single fixation that lasts too little or too long.

### 5.2. Analysis of the Saccade Duration

**Figure 6:**
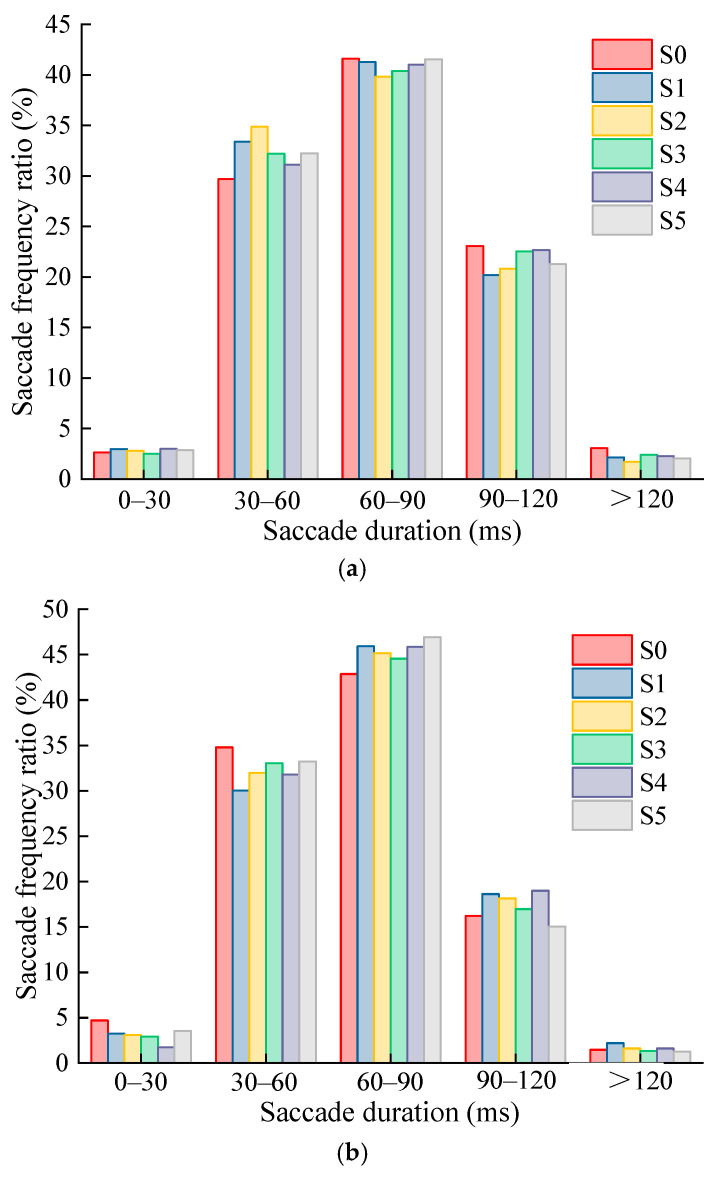

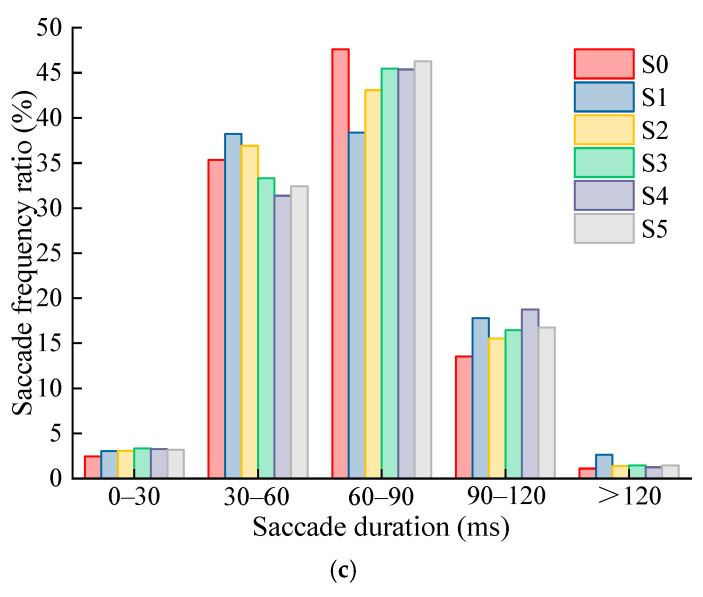
Distribution of driver’s saccade durations at different TSIV of the three-speed conditions. (**a**) 60 km/h; (**b**) 80 km/h; (**c**) 100 km/h.

The saccade duration of the participants at three speeds of 60 km/h, 80 km/h, and 100 km/h was counted by an interval time period of 30 ms. Figure 6 shows the statistical results of the saccade duration and saccade frequency ratio when the driver recognizes the various levels of TSIV under the three-speed conditions, respectively.

The following findings can be drawn from counting the participants’ saccade frequency ratios over each time period at the three different speeds of 60 km/h, 80 km/h, and 100 km/h.

(1) Under the three-speed conditions, the change law of the saccade frequency in each saccade duration interval is highly constant when the driver identifies the various levels of TSIV. The fraction exhibits a tendency of initially growing and then declining with an increase in saccade duration.
(2) The single saccade duration of drivers in the three-speed conditions, when they recognize the different levels of TSIV, is mainly concentrated in the time period of 30–120 ms, accounting for more than 90% of the saccade frequency. The highest percentage of single saccade occurs in the 60–90 ms time frame, followed by 30–60 ms and 90–120 ms, respectively, suggesting that the duration of saccade behaviors when drivers are searching for information is generally not excessively long.

### 5.3. Analysis of the Saccade Amplitude

**Figure 7:**
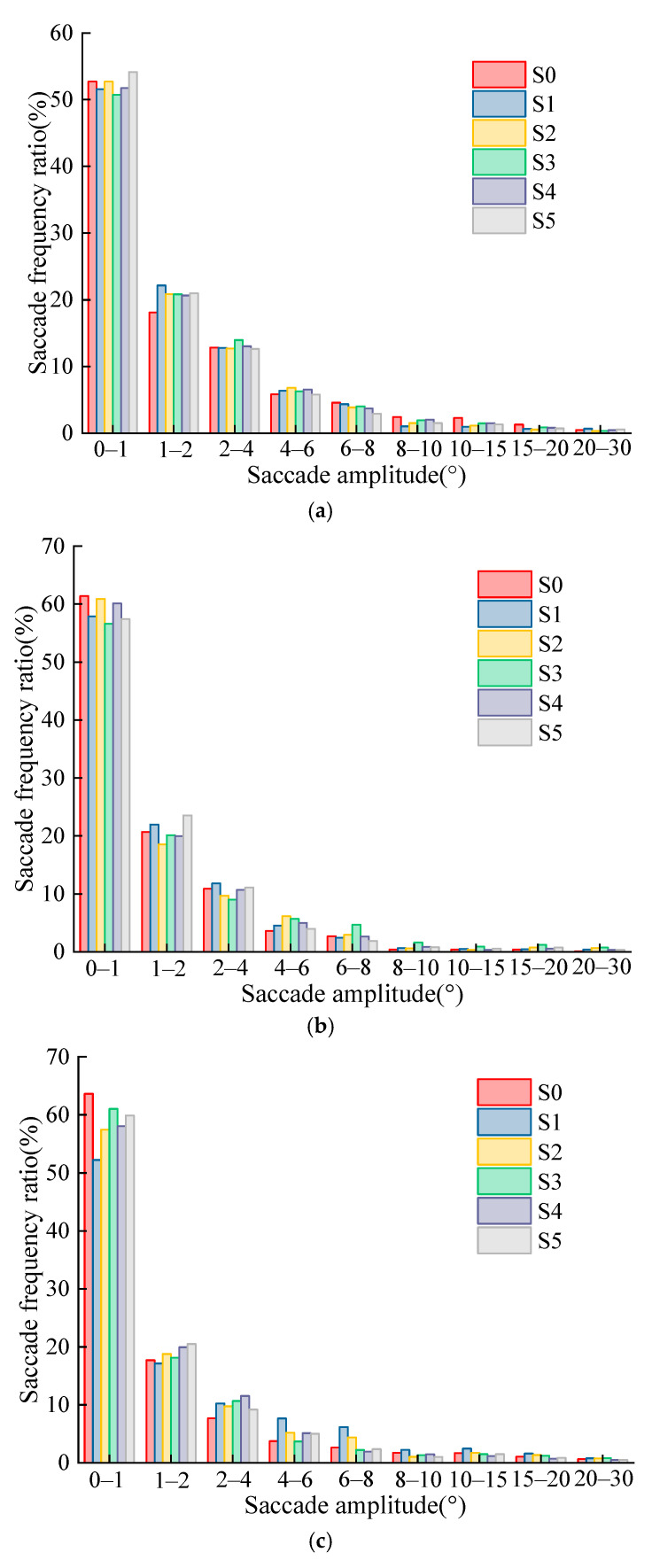
Distribution of driver’s saccade amplitude at different TSIV of the three-speed conditions. (**a**) 60 km/h; (**b**) 80 km/h; (**c**) 100 km/h.

The saccade amplitude of the participants at three speeds of 60 km/h, 80 km/h, and 100 km/h was counted by an interval time period. Figure 7 shows the statistical results of the saccade amplitude and saccade frequency ratio when the driver recognizes the various levels of TSIV under the three-speed conditions, respectively.

By analyzing the driver’s saccade amplitude at 60 km/h, 80 km/h, and 100 km/h, the following results can be identified.

(1) Under the three-speed conditions, when the driver recognizes the TSIV at different levels, the change law of the proportion of the saccade amplitude in each section is very consistent. With the increase of the saccade amplitude, the proportion gradually decreases and eventually stabilizes.
(2) The single saccade amplitude of the driver when recognizing the TSIV at different levels under the three-speed conditions is mainly concentrated in the range of 0°–4°, accounting for more than 80%. Among them, the occurrence frequency of a single saccade in the range of 0°–1° is the highest, as high as more than 50%, followed by 1°–2° and 2°–4°, accounting for about 20% and 10%, respectively. In addition, the percentage of saccade amplitude greater than 8° is very small. In general, the saccade behavior of drivers when searching and obtaining information is mainly the small saccade amplitude behavior, and the large saccade amplitude behavior rarely occurs.

## 6. Discussion

### 6.1. Differences in the Average Fixation Duration

**Figure 8:**
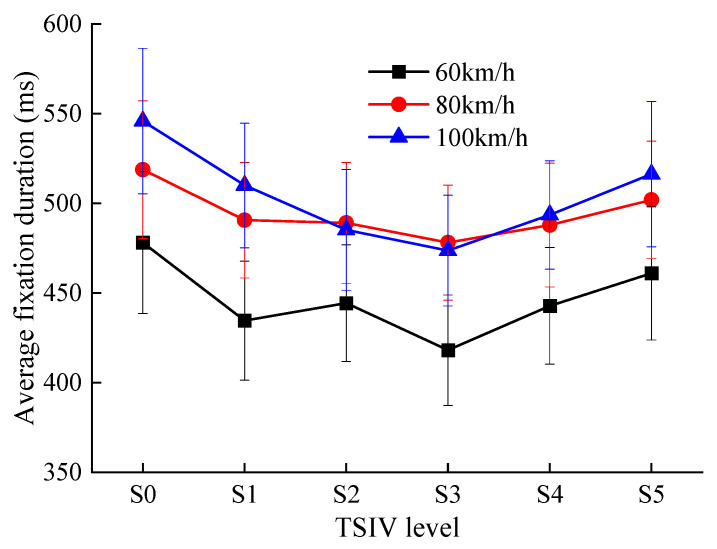
The comparison of average fixation duration for each TSIV at the three speeds.

The trend in the average fixation duration of drivers when recognizing different levels of TSIV at the three-speed conditions is shown in Figure 8.

Combined with the previous analysis, the following results can be obtained from Figure 8.

(1) When drivers recognize different levels of TSIV under the three-speed conditions, the average fixation duration of the drivers changes in a manner that is essentially similar. They have a pattern of first reducing and then increasing as TSIV rises. Additionally, the S3 TSIV level has the shortest average fixation duration of drivers in all three-speed conditions. This shows that the average fixation duration of drivers is greatly impacted by the fluctuation in the TSIV. On roads with insufficient or excessive TSIV, drivers show longer average fixation duration, indicating that they are more mentally and visually taxed under these circumstances and are less able to gather and process pertinent information. They also have more trouble processing the intended information [34,35].
(2) The average fixation duration of drivers in all three-speed conditions is the smallest in the S3 TSIV level, and the standard deviation of the average fixation duration in the S3 TSIV level is smaller than in the other information conditions, which also indicates that drivers in the S3 TSIV level are more capable of perceiving and acquiring information about the road traffic environment, can process the target information efficiently, have better visual coordination and less visual load, and this state is conducive to drivers ensuring normal and safe driving [36].
(3) The driver’s average fixation duration varies from 418 ms to 478 ms at 60 km/h, 478 ms to 518 ms at 80 km/h, and 473 ms to 546 ms at 100 km/h. It can be found that the value and range of average fixation durations become significantly longer as the speed increases for the same TSIV level. This shows that with the increase in speed, the driver’s ability to perceive and acquire information about the road traffic environment begins to decrease. The driver’s dynamic field of vision becomes smaller as the driving speed increases, that is, the area of the useful field of vision becomes smaller, which results in a smaller recognition and visual search range for the driver as well [27]. At the same time, with the increasing speed, the density and frequency of road traffic environment information also increase sharply, which requires the driver to identify and process more information in a shorter period of time. As a result, it is more difficult for drivers to process information [35]. The average fixation duration and its range of drivers are significantly increased in order to ensure that sufficient valid traffic information is obtained [37].

### 6.2. Differences in the Average Fixation Duration

**Figure 9:**
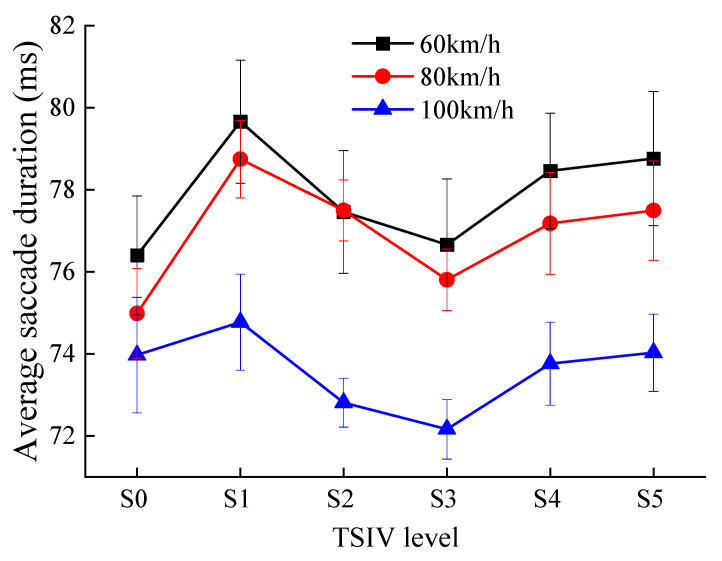
The comparison of average saccade duration for each TSIV at the three speeds.

The trend in the average saccade duration of drivers when recognizing different levels of TSIV at the three-speed conditions is shown in Figure 9.

Combined with the previous analysis, the following results can be obtained from Figure 9.

(1) When drivers recognize various levels of TSIV under the three-speed conditions, the changing trend of the average saccade duration of the drivers is essentially the same. They exhibit the tendency of initially growing, then declining, and finally climbing once again as TSIV increases. Additionally, in a TSIV-enabled road environment, drivers’ average saccade time under three-speed conditions is shortest at the S3 TSIV level. This suggests that changes in TSIV have a significant impact on changes in the average saccade duration of drivers. The average saccade duration of drivers is higher on roads with too little and too much TSIV, reflecting that drivers spend more time searching for target information, as well as reflecting the complexity of drivers in processing information, the longer saccade duration means that drivers are less able to recognize useful information [38].
(2) Under speeds of 60 km/h and 80 km/h, the drivers’ average saccade duration is shorter in the S0 TSIV level than in the other TSIV levels, suggesting that drivers may be visually distracted as a result of the monotonous road traffic environment and weak information stimulus. In other words, when this happens, the drivers’ visual attentiveness is low, making it challenging for them to react to and handle crises as they arise in a timely manner, perhaps endangering their safety. The average saccade duration of a motorist who is speeding, however, is much shorter at all TSIV levels than it is in the other two speed conditions, and it is not minimized at the S0 level. This is because the increase in speed places a higher demand on the driver’s visual search, and the driver’s average saccade duration increases slightly as a result of the increased psychological stress caused by driving at high speeds on roads without TSIV [27]. However, overall, the average saccade duration of drivers in the S3 TSIV level is lower and less discrete compared to the other levels with TSIV. This indicates that drivers are more efficient in searching for information about the road traffic environment under this condition, and are able to search for useful information quickly and recognize the target information better.
(3) The driver’s average saccade duration varies from 76.4 ms to 79.6 ms at 60 km/h, 74.9 ms to 78.7 ms at 80 km/h, and 72.2 ms to 74.8 ms at 100 km/h. It can be found that for the same TSIV level, although there is no significant difference between the average saccade duration of drivers at 60 km/h and 80 km/h, it is clear from the trend in the data that the average saccade duration and its range of variation tend to become smaller as the speed increases. This indicates that the driver spends significantly less time searching for information as the speed increases due to the fact that the increase in speed directly makes the driver’s dynamic field of view and spatial recognition range smaller and the driver’s search for information more difficult.

### 6.3. Differences in the Average Saccade Amplitude

**Figure 10:**
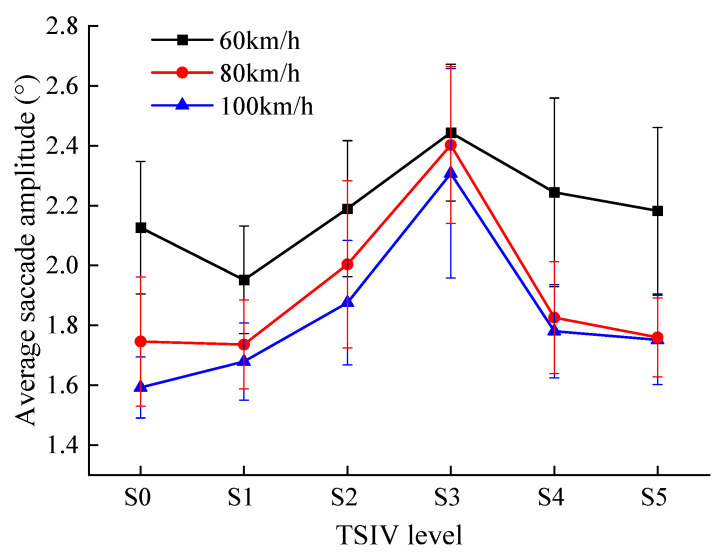
The comparison of average saccade amplitude for each TSIV at the three speeds.

The trend in the average saccade amplitude of drivers when recognizing different levels of TSIV at the three-speed conditions is shown in Figure 10.

Combined with the previous analysis, the following results can be obtained from Figure 10.

(1) When drivers recognize various levels of TSIV under the three-speed conditions, the changing trend of the average saccade amplitude is roughly the same. With an increase in TSIV, they display a trend of initially growing and then declining. Additionally, the average saccade amplitude of drivers under the three-speed conditions is the maximum threshold under the S3 TSIV level. This suggests that changes in TSIV have a significant effect on the change in drivers’ average saccade amplitude, with both too little and too much TSIV resulting in decreased average saccade amplitudes. This reflects the limited information that can be obtained with each fixation during the driver’s visual search under conditions of TSIV insufficient and overload, resulting in smaller subsequent saccade distances [39], as well as the greater difficulty for the driver to extract information, which makes the saccade amplitude smaller.
(2) The depth of attention of the driver when searching for information while driving can be accurately measured by the saccade amplitude. The average driver’s saccade amplitude at the three speeds is highest in the S3 TSIV level, and the standard deviation is lowest compared to the other levels, showing that the driver can obtain more about the road traffic environment at each fixation in the S3 TSIV level, making it easier for the driver to observe and comprehend the surrounding traffic environment and lowering the drowsiness, improving driving safety.
(3) The driver’s average saccade amplitude varies from 1.95° to 2.44° at 60 km/h, 1.59° to 2.31° at 80 km/h, and 1.74° to 2.40° at 100 km/h. It can be found that the average saccade becomes significantly shorter as the speed increases for the same TSIV level. This is due to the fact that the driver’s dynamic visual field and spatial recognition range become smaller with the increase in driving speed, and it is difficult for the driver to obtain enough useful traffic environment information by a single fixation, which leads to the smaller following saccade amplitude, and the driver will show obvious tension at this time [40].

### 6.4. Analysis of the Visual Workload Intensity

From the above analysis, it can be seen that the three indicators of fixation duration, saccade duration, and saccade amplitude are all effective in characterizing the driver’s visual workload on the TSIV [41]. Therefore, these three eye movement indicators can be combined to form a comprehensive indicator of the driver’s visual workload.

In this paper, the method of factor analysis is used to determine the weight of each indicator, and the factor score coefficient can be used as the weight value of the indicator to evaluate its importance. The factor scores are shown in Table 7.

We can derive the relational equation of the comprehensive indicator characterizing the driver’s visual workload intensity from Table 7. The coefficient of the relational equation is the above factor score. Additionally, according to the previous analysis, it can be seen that the driver’s visual workload intensity is positively correlated with fixation duration and saccade duration and negatively correlated with saccade amplitude, so the expression of the driver’s visual workload intensity can be expressed as:

F = 0.034FD + 0.558SD − 0.559SR,(11)

where: F is the driver’s visual workload intensity (dimensionless), FD is the driver’s fixation duration (ms), SD is the driver’s saccade duration (ms), SR is the driver’s saccade amplitude (°).

**Figure 11:**
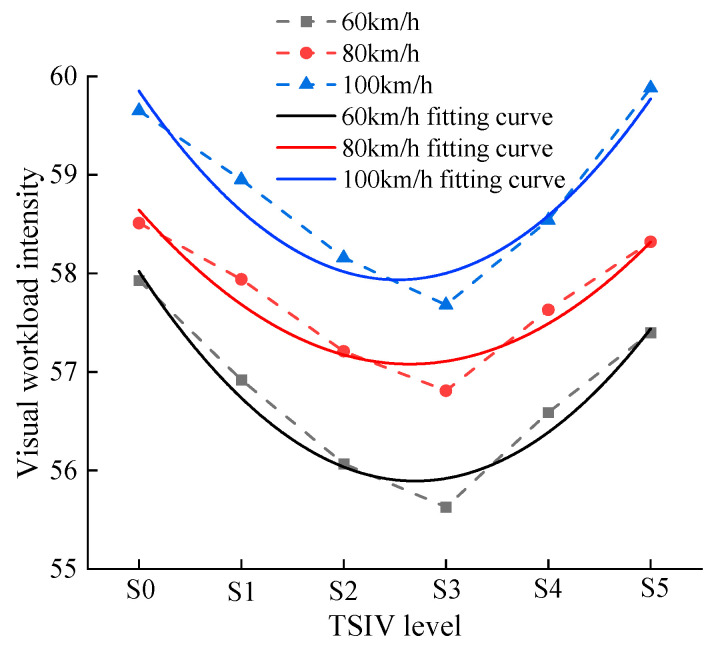
Visual workload intensity of the driver when recognizing different levels of TSIV under three speed conditions.

The visual workload intensity was calculated when the participants recognized different levels of TSIV at 60 km/h, 80 km/h, and 100 km/h speeds, as shown in Figure 11. Additionally, the relationship between visual workload intensity and TSIV was analyzed. The results and ANOVA of the fit of the driver’s visual workload intensity to the TSIV are shown in Table 8 and Table 9.

According to the above fitting results, the non-linear fitting equations of the driver’s visual workload intensity and TSIV under the three-speed conditions of 60 km/h, 80 km/h, and 100 km/h are $y=58.02-0.16x+0.003x^{2}$, $y=58.64-0.12x+0.002x^{2}$, and $y=59.85-0.15x+0.003x^{2}$, respectively. The fitting determination coefficients *R*^2^ are 0.92, 0.85, and 0.87, respectively. It shows that the reliability of the fitted model is good, indicating that the driver’s visual workload intensity and the TSIV of different levels in the three-speed conditions are well binomially distributed. In addition, the results of the fitted variance show that the *p*-values are all less than 0.05, which also indicates that the correlation is statistically significant.

From the above statistical analysis, it is clear that:

(1) The changing trend of the visual workload intensity of drivers is roughly the same when drivers recognize different levels of TSIV under the three-speed conditions. They show the trend of decreasing first and then increasing with the increase of TSIV. This shows that the TSIV has a significant influence on the visual workload intensity of drivers.
(2) The visual workload intensity of the driver is the lowest under the S3 TSIV level under the three-speed conditions, indicating that the driver’s psychological pressure is the lowest when driving under the S3 TSIV level, and the driving workload is lower, which is conducive to ensuring the driver’s driving safety.
(3) At the same TSIV level, the driver’s visual workload intensity increases with the increasing speed.

## 7. Limitations and Directions for Future Research

To further study the impact of TSIV on traffic safety, the following issues need to be further discussed:

(1) Drivers of different genders, ages, driving styles, and driving experience may have different perceptions and performances on the TSIV, so further research needs to consider different types of drivers.
(2) Different requirements for TSIV may also apply to different types of roads, including freeways, urban expressways, and tunnels. Therefore, different types of roads need to be specifically studied in additional research.
(3) This paper only analyzes the visual characteristics and visual workload of drivers. In the future, the impact of the TSIV on the driver’s perception and behavior can be further analyzed by combining it with data from the driver’s EEG, ECG, driving behavior, and vehicle operating status.
(4) The number of participants recruited for the tests was 30, and the number was limited. To obtain more accurate test results, it is suggested that the number of subjects should be appropriately expanded in future studies.

## 8. Conclusions

In this study, simulated driving tests were conducted to obtain eye movement data of participants when recognizing different levels of TSIV and analyzed the influence of TSIV on the visual characteristics and visual workload of drivers, and then the following conclusions were obtained:

(1) Three indicators—fixation duration, saccade duration, and saccade amplitude—were chosen by PCA as sensitive indicators of the driver’s eye movement behavior influenced by the TSIV while driving.
(2) When drivers recognize traffic signs, the duration of a single fixation is mainly concentrated in the time period of 50–400 ms, and the proportion of fixation frequency accounts for more than 70%. The duration of a single saccade is mainly concentrated in the time period of 30–120 ms, with the proportion single saccade accounting for more than 90%. The amplitude of a single saccade is mainly concentrated in the range of 0°–4°, accounting for more than 80% of the saccades.
(3) The average fixation duration exhibits a trend of decreasing first, then increasing, and the average saccade duration displays a trend of increasing first, then decreasing, and then increasing again, and the average saccade amplitude shows a trend of increasing first, then decreasing. These trends are all correlated with the increase in TSIV. Drivers have the shortest average fixation duration, the shortest average saccade duration, and the greatest average saccade amplitude at the S3 TSIV level (30 bits/km), and drivers have the best visual performance in this condition.
(4) With the increase of TSIV, the driver’s visual workload intensity shows a trend of decreasing first and then increasing and is lowest at the S3 TSIV level. It shows that drivers have the lowest psychological stress and lower driving workload when driving at the S3 TSIV level, which is conducive to ensuring the driver’s safety when driving in this condition.
(5) The setting of traffic signs in the experiment of this paper is based on the current standards of China, and the form of traffic signs is of Chinese characteristics. Therefore, the conclusions drawn in this paper are only applicable to the suggestion of proposed TSIV on highways in China. This paper finds that reasonable and appropriate TSIV plays an important role in traffic engineering and driving safety. The research conclusion can provide a theoretical basis for the setting and improvement of highway traffic signs in China.

## Figures and Tables

**Table 1:** Information volume and weight of each traffic sign information element.

Information Elements	Information Volume (Bits)	Weight
Chinese character	11.8	0.25
English character	4.7	0.06
Arabic numeral	3.3	0.15
Geometric figure	2.6	0.11
Color	3.6	0.12
Pointing symbol	4.9	0.22
Graphic symbol	5.6	0.09

**Table 2:** The levels of the TSIV in the test scenarios.

Level	S0	S1	S2	S3	S4	S5
Information volume (bits/km)	0	10	20	30	40	50

**Table 3:** Correlation matrix of eye movement indicators.

	Indicators	BD	FD	PD	SD	SA	SPV	SAV
**Correlation coefficients**	**BD**	1.000	0.531	0.250	−0.151	−0.350	−0.255	−0.227
	**FD**	0.531	1.000	0.128	−0.084	−0.410	−0.256	−0.348
	**PD**	0.250	0.128	1.000	0.019	−0.629	−0.589	−0.594
	**SD**	−0.151	−0.084	0.019	1.000	0.238	−0.192	−0.378
	**SA**	−0.350	−0.410	−0.629	0.238	1.000	0.875	0.801
	**SPV**	−0.255	−0.256	−0.589	−0.192	0.875	1.000	0.951
	**SAV**	−0.227	−0.348	−0.594	−0.378	0.801	0.951	1.000
***p*** **-value for significance test**	**BD**		0.008	0.144	0.263	0.065	0.139	0.168
	**FD**	0.008		0.296	0.363	0.036	0.138	0.066
	**PD**	0.144	0.296		0.468	0.001	0.003	0.003
	**SD**	0.263	0.363	0.468		0.156	0.208	0.050
	**SA**	0.065	0.036	0.001	0.156		0.000	0.000
	**SPV**	0.139	0.138	0.003	0.208	0.000		0.000
	**SAV**	0.168	0.066	0.003	0.050	0.000	0.000	

**Table 4:** KMO test and Barlett Test of Sphericity for eye movement indicators.

Kaiser–Meyer–Olkin Statistic		0.548
Barlett Test of Sphericity	χ2	137.480
	df	21
	Sig.	0.000

**Table 5:** Cumulative contribution rates of variance of eye movement indicators.

Components	Initial Eigenvalues			Contribution Rates of Variance		
	Eigenroots	Contribution Rates of Variance (%)	Cumulative Contribution Rates (%)	Eigenroots	Contribution Rates of Variance (%)	Cumulative Contribution Rates (%)
1	3.558	50.825	50.825	3.558	50.825	50.825
2	1.459	20.846	71.671	1.459	20.846	71.671
3	0.991	14.154	85.825	0.991	14.154	85.825
4	0.576	8.232	94.056			
5	0.387	5.535	99.591			
6	0.022	0.313	99.904			
7	0.007	0.096	100.000			

**Table 6:** Matrix of eye movement indicators components.

Indicator	Components		
	1	2	3
BD	−0.469	0.618	0.358
FD	−0.497	0.547	0.807
PD	−0.525	−0.114	0.632
SD	−0.082	−0.857	−0.321
SA	0.959	−0.134	0.293
SPV	0.727	0.251	0.075
SAV	0.623	0.334	−0.110

**Table 7:** Factor score coefficient.

Eye Movement Indicators	Factor Score
Fixation duration	0.034
Saccade duration	0.558
Saccade amplitude	0.559

**Table 8:** Fitting results of driver’s visual workload intensity and TSIV under three-speed conditions.

Speeds	Intercept		B1		B2		Statistics
	Value	Standard Error	Value	Standard Error	Value	Standard Error	Adj. R-Square
60 km/h	58.02	0.216	−0.16	0.021	0.003	3.89148 × 10^−4^	0.92
80 km/h	58.64	0.229	−0.12	0.022	0.002	4.14849 × 10^−4^	0.85
100 km/h	59.85	0.276	−0.15	0.026	0.003	4.98139 × 10^−4^	0.87

**Table 9:** ANOVA for fitting of driver’s visual workload intensity and TSIV under three-speed conditions.

Speeds	Parameter	DF	Sum of Squares	Mean Square	F Value	Prob > F
60 km/h	Model	2	3.416	1.708	30.21	0.01
	Error	3	0.169	0.057		
	Total	5	3.586			
80 km/h	Model	2	1.934	0.967	15.05	0.027
	Error	3	0.193	0.064		
	Total	5	2.127			
100 km/h	Model	2	3.364	1.682	18.16	0.021
	Error	3	0.278	0.093		
	Total	5	3.642			

## Data Availability

Data generated in this study are available upon request.
